# Remote Skin Cancer Diagnosis: Adding Images to Electronic Referrals Is More Efficient Than Wait-Listing for a Nurse-Led Imaging Clinic

**DOI:** 10.3390/cancers13225828

**Published:** 2021-11-20

**Authors:** Leah Jones, Michael Jameson, Amanda Oakley

**Affiliations:** 1 Waikato District Health Board, Hamilton 3204, New Zealand; michael.jameson@waikatodhb.health.nz (M.J.); amanda.oakley@waikatodhb.health.nz (A.O.); 2 Waikato Clinical Campus, University of Auckland, Hamilton 3204, New Zealand

**Keywords:** teledermatology, teledermoscopy, skin cancer, squamous cell carcinoma, basal cell carcinoma, melanoma, telemedicine, skin neoplasms, referral and consultation

## Abstract

**Simple Summary:**

Skin cancer is a significant cause of death and disability, particularly in New Zealand. Expert diagnosis reduces unnecessary excision of benign lesions, reduces patient anxiety, and allows early identification of skin cancer, particularly of melanoma. The study assessed an electronic referral pathway for teledermatology—diagnosing skin lesions remotely using a standardised template with regional, close-up, and dermoscopic images—and compared this to scheduled nurse-led teledermoscopy clinics. A dermatology opinion was reached more rapidly with comparable efficacy when referrals include good quality images, compared to nurse-led imaging clinics.

**Abstract:**

We undertook a retrospective comparison of two teledermatology pathways that provide diagnostic and management advice for suspected skin cancers, to evaluate the time from referral to diagnosis and its concordance with histology. Primary Care doctors could refer patients to either the Virtual Lesion Clinic (VLC), a nurse-led community teledermoscopy clinic or, more recently, to the Suspected Skin Cancer (SSC) pathway, which requires them to attach regional, close-up, and dermoscopic images. The primary objective of this study was to determine the comparative time course between the SSC pathway and VLC. Secondary objectives included comparative diagnostic concordance, skin lesion classification, and evaluation of missed skin lesions during subsequent follow-up. VLC referrals from July to December 2016 and 2020 were compared to SSC referrals from July to December 2020. 408 patients with 682 lesions in the VLC cohort were compared with 480 patients with 548 lesions from the 2020 SSC cohort, matched for age, sex, and ethnicity, including histology where available. Median time (SD) from referral to receipt of teledermatology advice was four (2.8) days and 50 (43.0) days for the SSC and VLC cohorts, respectively (*p* < 0.001). Diagnostic concordance between teledermatologist and histopathologist for benign versus malignant lesions was 70% for 114 lesions in the SSC cohort, comparable to the VLC cohort (71% of 122 lesions). Referrals from primary care, where skin lesions were imaged with variable devices and quality resulted in faster specialist advice with similar diagnostic performance compared to high-quality imaging at nurse-led specialist dermoscopy clinics.

## 1. Introduction

Skin cancer prevalence is increasing in association with an ageing population with sun-damaged skin [1]. New Zealand has one of the highest rates of melanoma in the world, due to high ultraviolet intensities in the Southern Hemisphere during summer and high proportion of the population with fair skin [2,3]. It has an age-standardised incidence of 30–50 per 100,000 [4,5]. The incidence of basal cell carcinoma (BCC) and squamous cell carcinoma (SCC) is harder to evaluate, given that these are not required to be reported to the cancer registry; however, research has estimated the incidence of cutaneous SCC to be 425–668 per 100,000 and BCC to be 1177 per 10,000, putting it among the highest in the world [6,7]. New Zealand has limited access to publicly-funded dermatology services, with one dermatologist per 274,146 population, far less than the recommended 1 per 100,000 based on referral numbers [8]. In 2016 and 2020 respectively, the Waikato District Health Board, a tertiary dermatology referral centre, had 2.5 and 2.0 full-time equivalent dermatologists for a population of about half a million people [9].

Teledermatology aims to increase access to skin lesion assessment by a dermatologist by using telecommunications technology to deliver healthcare. There are two main methods of teledermatology: video conferencing, in which the patient is reviewed virtually in real-time by a specialist and ‘store and forward’, where the images are taken to be reviewed at a later time. Advances in technology have increased the opportunities available in teledermatology. It has been gaining in popularity, particularly in light of the COVID-19 pandemic and subsequent restrictions to in-person consults. Recent surveys of Dermatologists in the United States and India showed more than 80% were now offering teledermatology [10]. Our earlier research confirmed that skin tumours could be accurately diagnosed by teledermoscopy [11], which led in January 2010 to the introduction of a store-and-forward method of teledermatology we called the Virtual Lesion Clinic (VLC) [12]. General practitioners (GPs) refer patients with one or more skin lesions of concern to the dermatology service. Patients are allocated to a nurse-led imaging clinic in one of three locations. After reviewing the files, the teledermatologist discharges the patient or refers them to a specialist surgical service for excision, arranges to monitor the lesion at the VLC, or advises the GP on treatment in primary care. By mid-2019, the publicly funded VLC had evaluated more than 11,000 lesions in 6600 patients attending scheduled nurse-specialist clinics in three community locations [13]. The safety and efficacy of the VLC model has been confirmed [14,15].

The introduction of electronic referrals in February 2016 led to a direct Suspected Skin Cancer (SSC) advice service in July 2017. The SSC pathway template expects the referring Primary Care doctor to attach regional, close-up, and dermoscopy images for 1 to 5 lesions. This has become possible mainly due to the ubiquitous use of smartphones with adequate cameras and dermoscopic attachments [16]. The teledermatologist provides advice to the GP directly. Unlike VLC, the SSC pathway requires the GP to arrange skin lesion excision independently. If images are inadequate for diagnosis, this is conveyed to the GP with a request for further images or to request assessment at the VLC. This SSC template is currently used for more than half of the referrals to Dermatology each month (the remainder use the ‘General Dermatology’ template).

The benefit of increased access to skin cancer screening by teledermoscopy, particularly in screening rural and low-income groups, has been well established [17,18]. A 2018 systematic review that included 16 studies, predominantly cross-sectional and observational, reported diagnostic accuracy in eight studies [19]. This included our local interventional study, which compared dermatologist concordance of teledermoscopy and in-person reviews, where the concordance between dermatologists was 74% [11]. Teledermoscopy diagnosis of 43 skin lesions was concordant with histology in 85% of cases compared to 91% for in-person diagnosis [20].

An interventional study of store-and-forward teledermoscopy compared to previous in-person referrals found the time to diagnose 79 patients reduced from 70 days to 0.5 days (*p* < 0.001) and reduced the time until definitive treatment from 73.5 days to 3.0 days (*p* < 0.001) [21]. The teledermatology images were assessed by two dermatologists independently with at least partial concordance in all cases (complete concordance in 32%) [21]. Of the 29 patients that went on to have in-person consultation, the concordance between teledermatologist and dermatologist was partially concordant in 15 patients (52%) and completely concordant in 11 patients (38%) [21]. A retrospective cohort study of 2385 referrals in a poorly-serviced area in the USA showed an 84% reduction in in-person consultations and reduced wait time from 77 days to 28 days by using teledermatology [22].

The objectives of this retrospective study were to assess the efficacy and efficiency of direct teledermoscopy via GP electronic referral to the SSC pathway in comparison to the VLC pathway. The primary objective was to compare the time from receipt of the referral to return of the Dermatologist advice. Secondary objectives were, firstly, to determine diagnostic concordance between GP and dermatologist (and histology if available) between the SSC 2020 cohort and VLC cohorts from 2020 and 2016, and secondly, the identification of any incidental suspected skin cancers found during follow-up specialist appointments.

## 2. Materials and Methods

The Health and Disabilities Committee (HDEC) of New Zealand determined the study to be a service review and therefore out of scope for formal ethics approval (22 December 2020).

We undertook a retrospective review to compare outcomes of referrals for possible skin cancer to either the VLC or SSC pathway in 2020 or the VLC in 2016. As there was a reduction in referral numbers during the first four months of 2020, due to healthcare interruptions related to COVID-19, we chose to evaluate referrals received 1 July–31 December in 2016 and 2020 for consistency.

Referrals were identified by searching departmental records. For the 2020 SSC and VLC cohorts, the keywords ‘lesion’ and ‘skin cancer’ were used; for the 2016 VLC cohort a unique coding identifier was used. Referrals for reasons other than a suspected skin cancer were excluded.

From the 2020 SSC referrals, 2020 VLC and 2016 VLC referrals, we determined the number of referrals, the time from referral to advice, the number of lesions per referral, and patient demographics—including age, sex, ethnicity, and whether urban, rural, or semi-rural residence—based on the Statistics New Zealand Urban Accessibility Classification [23]. For the SSC pathway cohort, we recorded data on image quality. Propensity score matching was used to create a subset of the 2020 SSC pathway cohort matched for age, gender, and ethnicity to those in the 2016 VLC cohort. This was used to obtain data on time to definitive management, lesion histology, and additional lesions of concern during specialist review.

We classified skin lesions as benign, malignant, or pre-malignant, recorded the specific diagnosis, and determined the diagnostic concordance between GP, dermatologist, and histology report, along with the management advice, the time to definitive treatment, and the number of incidental lesions identified at related in-person specialist appointments (Appendix A).

For the benign/malignant classification, a diagnosis was concordant if the GP or teledermatologist’s diagnosis (or in the case of multiple diagnoses, the first diagnosis) was classified in the same group (benign, malignant, pre-malignant) as histology where available, otherwise in the same group as the teledermatologist’s diagnosis in the case of GP-dermatologist concordance. For the specific diagnosis, this was deemed completely concordant if the GP or teledermatologist only provided one diagnosis and this was the same as the histology diagnosis. A diagnosis was partially concordant if more than one diagnosis was provided by the GP or teledermatologist and one of those was the same as the histology. If a diagnosis was not provided or was labelled uncertain by the GP or teledermatologist, this was deemed not concordant.

Statistical analysis was performed using Microsoft Excel (2016, version 16.0.4591.1000, Microsoft, Redmond, WA, USA) and IBM SPSS Statistics (version 26, IBM, Armonk, NY, USA) software. The chi-squared test was used for comparison of categorical variables between groups. One-way analysis of variance (ANOVA) was used for comparison of continuous variables. A two-sided *p*-value of < 0.05 was used to determine statistical significance.

## 3. Results

**Figure 1:**
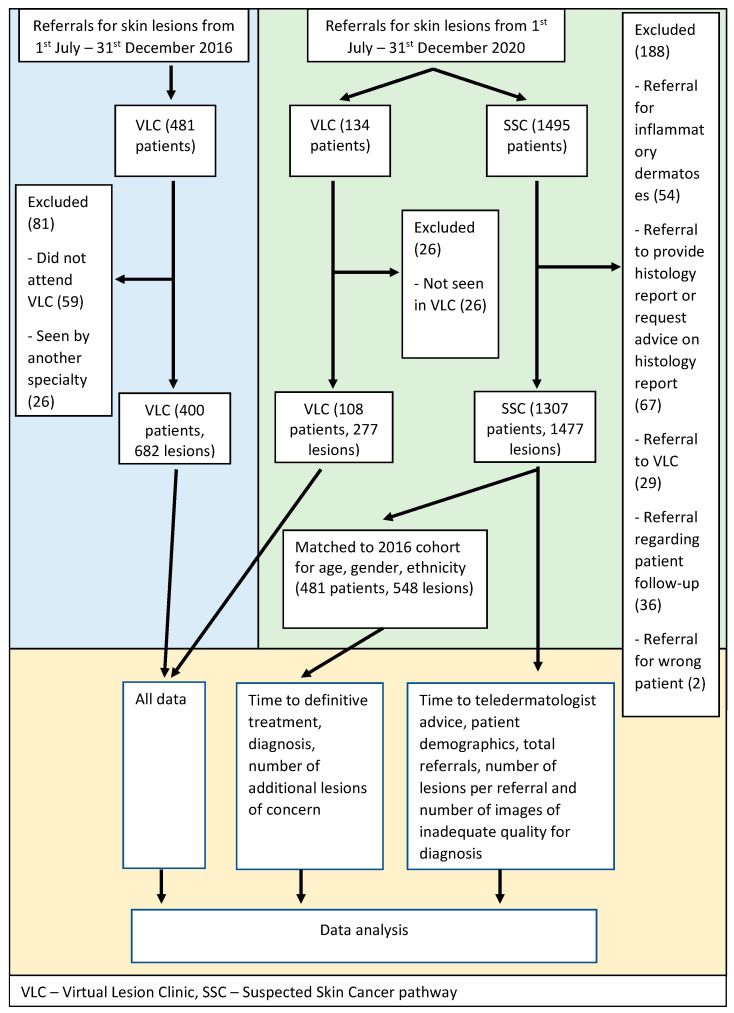
Flow chart.

Between 1 July and 31 December 2016, 481 patients were referred to the VLC, of whom 400 patients with 682 lesions were eligible for analysis. In the period 1 July to 31 December 2020, 1307 patients of 1495 referred to the SSC pathway, and 108 patients (with 277 lesions) of 134 referred to the VLC, were eligible for analysis (Figure 1). 1307 met the eligibility criteria. Propensity score matching of the SSC cohort to the 2016 VLC cohort identified 481 patients with 548 lesions from the SSC cohort. Patient characteristics are detailed in Table 1.

Patients in the total SSC group had a mean age of 61 years compared to 59 years for the 2020 VLC group and 55 years for the 2016 VLC group (*p* < 0.001) (Table 1). There was a slight female preponderance (56%, 59%, and 64% respectively). Ethnicity was predominantly Caucasian with 84% New Zealand European in the SSC pathway total cohort, 78% in the SSC matched cohort, 74% in 2020 VLC cohort and 79% in 2016 VLC group. Maori ethnicity was claimed by 6%, 11% and 7% respectively. Analysis of patient and referrer location revealed a similar proportion of rural patients in all groups, however there was a larger portion of patients located semi-rurally in the 2020 VLC (60% for patients and 62% for referrers, *p* < 0.01) and 2016 VLC cohorts (42% for patients and 44% for referrers, *p* = 0.1, *p* = 0.02), compared to 37% for patients and 36% for referrers in the total SSC pathway group.

A group of 45 patients were lost to follow-up after receiving teledermatologist advice (19 (4%) in SSC matched cohort, 13 (12%) in the 2020 VLC cohort and 13 (3%) in the 2016 cohort). In the total SSC group, 71 lesions (5%) were unable to be diagnosed due to poor image quality. Another 115 lesions (9%) were able to be diagnosed but the images were noted by the dermatologist to be of poor quality (Table 2).

Diagnostic and management advice was provided by two teledermatologists in 2020 and by three in 2016 time period; one was common to both time periods. Median time from referral to teledermatologist advice was 4 days (range 0–19) in the SSC pathway total cohort. For the VLC cohorts, the median time to advice was 42 days (range 16–184) in 2020 and 50 days (range 17–313) in 2016 (*p* < 0.001) (Table 3). Waiting time for a clinic appointment was the rate-limiting factor, with a median time of 26 days (range 0–173) in 2020 and 43 days (range 1–308) in 2016. The time from advice to definitive treatment (e.g., excision, in-person review) was 21.5 days in the SSC pathway cohort compared to 45 days in the 2020 VLC cohort and 60 days in the 2016 cohort. This increases to 94 days and 112 days for the 2020 and 2016 VLC cohorts respectively when calculating time from acceptance of the referral to treatment.

**Figure 2:**
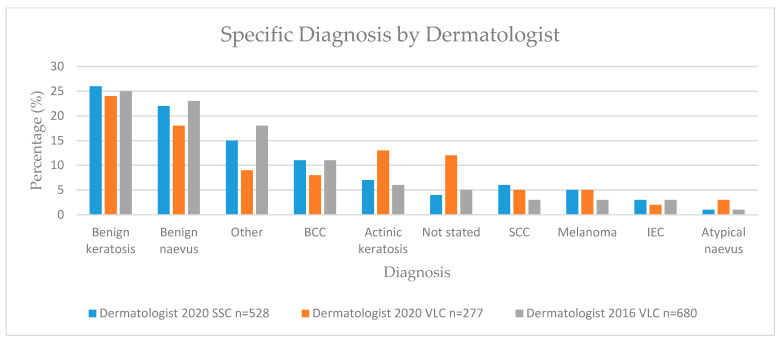
Specific lesion diagnosis by dermatologist. BCC—basal cell carcinoma; SCC—squamous cell carcinoma; IEC—intraepithelial carcinoma.

**Figure 3:**
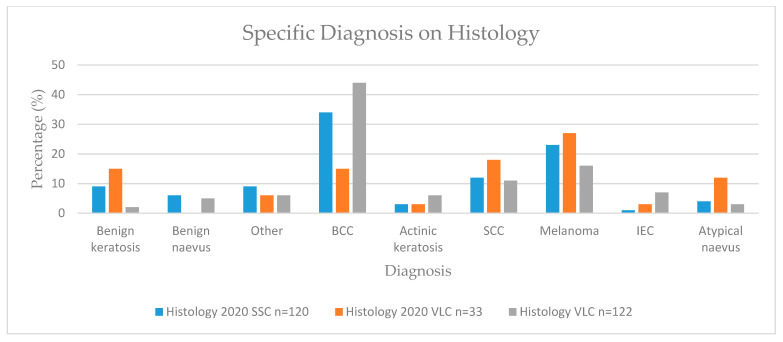
Specific lesion diagnosis on histology. BCC—basal cell carcinoma; SCC—squamous cell carcinoma; IEC—intraepithelial carcinoma.

Skin lesions were classified as benign or malignant and by specific diagnosis (Table 4, Figure 2 and Figure 3).

In the SSC pathway matched cohort, the teledermatologist recorded benign lesions in 65%, with a benign-to-malignant ratio of 3.0, compared to a benign-to-malignant ratio of 1.3 for GP (Table 4).

In the 2020 and 2016 VLC cohorts, the teledermatologist recorded benign lesions in 64% and 67%, and benign-to-malignant ratio of 3.4 and 3.8 respectively, compared to 1.7 and 0.8 for GP (Table 4).

Analysis of histology of malignant lesions revealed a keratinocytic-to-melanocytic ratio of 1.8 in the SSC matched cohort, 1.2 in the 2020 VLC cohort and 3.4 in the 2016 cohort (Table 5). Analysis of melanomas revealed a melanoma-in-situ-to-invasive melanoma ratio of 3.7 in the SSC matched cohort and 2.0 and 2.3 for the 2020 VLC and 2016 VLC cohorts respectively (Table 5).

No further management was recommended by the dermatologist for 56%, 57%, and 69% of matched SSC pathway, 2020 VLC and 2016 VLC cohorts respectively (Table 6). Surgical management with either biopsy or excision was recommended for 26% of lesions in the matched SSC group compared to 16% in the 2020 VLC and 16% in the 2016 VLC groups (*p* < 0.001). In-person review was more common in the VLC cohorts at 7% in 2020 and 5% in 2016, compared to 0% in the SSC pathway.

GP–dermatologist diagnostic concordance was available for 528 lesions in the SSC matched cohort, 172 lesions in the 2020 VLC cohort and 601 lesions in the 2016 VLC cohort (Table 7). The concordance for the benign/malignant classification was 58%, 22%, and 32% for the SSC, 2020 VLC and 2016 VLC cohorts, respectively (*p* < 0.001). Concordance for the specific diagnosis was 46% (35% complete, 11% partial), 31% (22% complete, 9% partial), and 24% (17% complete, 7% partial) for the SSC, 2020 VLC and 2016 VLC cohorts, respectively (*p* < 0.001).

Histology was available for 114 lesions in the SSC pathway matched cohort, 32 lesions in the 2020 VLC cohort, and 122 lesions in the 2016 cohort (Table 7).

For the SSC pathway cohort, dermatologist–histology concordance was 70% for benign/malignant classification, whereas GP–histology concordance was 60% (*p* = 0.01). Dermatologist–histology concordance for specific diagnosis was 66% (53% complete, 13% partial), compared to GP–histology concordance of 48% (40% complete, 8% partial) (*p* < 0.001).

For the 2020 VLC cohort, dermatologist–histology concordance was 63% for benign/malignant classification, compared to 12% for GP–histology concordance (*p* = 0.21). Dermatologist–histology concordance for specific diagnosis was 65% (59% complete, 6% partial), whereas GP–histology concordance was 12% (6% complete, 6% partial) (*p* = 0.73).

For the 2016 VLC cohort, dermatologist–histology concordance was 71% for benign/malignant classification compared to GP-histology concordance of 49% (*p* = 0.001). Dermatologist—histology concordance was 57% for specific diagnosis (57% complete, 0% partial) compared to GP-histology concordance of 46% (41% complete, 5% partial) respectively (*p* = 0.03).

The benign/malignant dermatologist–histology concordance was similar between the SSC pathway and 2016 VLC cohorts at 70% and 71% respectively. Non-concordant results for specific diagnoses were 34% in the SSC cohort and 43% in the 2016 VLC cohort (*p* < 0.001). There were 11 lesions overall (4%) that were identified as being likely benign by dermatologists and malignant by histology with four (4%) in the matched SSC pathway, two (6%) in 2020 VLC and five (4%) in 2016 VLC cohorts. The teledermatology recommendation was ‘no further management’ for two of these lesions, ‘monitoring’ for one lesion, ‘topical therapy’ for one lesion, ‘excision/biopsy to remove doubt’, and ‘in-person review’ for two lesions.

A public hospital specialist Dermatology or Plastic Surgery outpatient appointment for a study lesion was made for 156 patients (41 in the matched SSC pathway cohort, 24 in the 2020 VLC cohort and 91 in the 2016 cohort). A mean of 0.2 incidental lesions of concern were identified (0.2, 0.0, and 0.2 in SSC pathway, 2020 VLC and 2016 VLC cohorts, respectively) (Tables S1 and S2). Eight out of 11 lesions (73%) were malignant on histology in the SSC pathway cohort, with a keratinocytic to melanocytic ratio of 3.0. The 2016 VLC cohort was found to have 6 out of 16 lesions (38%) malignant and a keratinocytic to melanocytic ratio of 1.0.

The VLC nurse specialists identified 107 incidental lesions in patients attending imaging clinics (78 in 2016 and 29 in 2020). Histology was available for 26 lesions in the 2016 VLC cohort with a benign-to-malignant ratio of 0.2 and 11 lesions in the 2020 VLC cohort with a benign-to-malignant ratio of 0.0 (Table S3).

## 4. Discussion

Good quality teledermatology and teledermoscopy allow effective skin lesion diagnosis and management [24]. Electronic referrals from primary to secondary care are increasingly used by district health boards in New Zealand. Availability of outpatient appointments for patients with suspected skin cancer is outstripped by the number of referrals to dermatology; other sites may offer remote advice, hold dedicated clinics, or forward referrals to a surgical specialty. The use of teledermatology worldwide increased during 2020 due to the COVID-19 pandemic compared to few sites using it in 2016. We have compared GP referrals to our SSC pathway, where images of mixed quality were taken using various devices, to our nurse-led VLC, where uniformly high-quality images were taken with standardized cameras.

The study has met our primary objective to demonstrate a statistically significant reduction in time to dermatologist advice using the SSC pathway in 2020 compared to the VLC in 2016 and 2020, with a median time from referral to advice of 4 days compared to 42 days and 50 days, respectively (*p* < 0.001). The longer time to advice included waits due to missed or rescheduled appointments. This is comparable to a US teledermatology service, where the time to advice was reduced from 70 days when using in-person clinic assessments to 0.5 days when using electronic referrals [21]. There was also a reduction in time to definitive treatment when using the SSC pathway compared to the 2016 and 2020 VLC, both for time from referral assessment (or triage for patients referred to the VLC) and for the time from dermatologist advice to treatment. This is likely explained by the differences in how advice is managed in each pathway, with GPs arranging excisions in the SSC pathway. This can lead to excision of lesions of concern within a few days to weeks in the private sector. In contrast, lesions requiring excision identified at VLC are referred to plastic surgery or dermatology at the hospital with longer wait times. The Standards of Service Provision for Melanoma Patients in New Zealand (2014) recommended “patients referred with a high suspicion of melanoma receive their first cancer treatment within 62 days of receipt of referral” [25]. A large retrospective study by Conic et al. showed improved survival in patients treated within 90 days for melanoma stages I–III and improved survival for patients treated within 30 days for stage I melanoma [26]. The reduction in time to treatment by a median of 62.5 days and 90.5 days compared to 2020 VLC and 2016 VLC are likely to significantly improve the proportion of patients meeting these standards.

The higher proportions of melanoma-in-situ found in the SSC pathway matched cohort compared to the 2016 VLC cohort were striking. We suspect that the rapid response to referrals in the SSC pathway allowed GPs to get a second opinion on lesions they would have otherwise managed independently. The total referral numbers for 2020 SSC pathway were significantly higher than for the 2016 VLC and the GP benign-to-malignant ratio in the SSC pathway matched cohort was higher at 1.3 compared to 0.8 in the 2016 VLC. This also hints at a lower threshold for referral.

Interpretation is complicated by differing rates of diagnostic uncertainty in the referrals: the SSC pathway template required the GP to select a suspected diagnosis (including an option, ‘uncertain’ in 20%), but this was not required for referrals to the VLC in 2016 where any referral without a diagnosis was deemed uncertain (42%).

The histology benign-to-malignant ratio was higher in the SSC pathway matched cohort (0.6) than the 2016 VLC cohort (0.2). This could indicate that dermatologists were conservative in diagnosing SSC pathway lesions due to some images of inferior quality. It is important to note that both ratios are very low. A recent systematic review estimated the number-needed to biopsy to find one melanoma for global dermatologists to be 7.5 [27]. In our study, the number needed to biopsy for melanoma was 4.2 in the SSC pathway cohort and 6.1 in the 2016 VLC cohort, despite including keratinocytic skin cancers. This raises the concern of missed melanoma. The high ratio of melanoma-in-situ to melanoma indicating enhanced early diagnosis of melanoma through the SSC pathway, reduces this possibility. Conversely, given these higher rates of melanoma-in-situ in the 2020 SSC pathway cohort, we need to be mindful of the possibility of overdiagnosis. The natural history of melanoma-in-situ is still not completely established [28]. Studies with high melanoma-in-situ excision rates or those assessing skin cancer screening, have frequently failed to show a mortality benefit [28,29]. In addition, the considerable variability in histopathologist classification of melanoma-in-situ versus atypical naevus further complicates this assessment [30]. There is currently no consensus on the best way to manage the risk of overdiagnosis, whilst still ensuring early treatment of invasive melanoma. Perhaps better education in skin lesion assessment will lead to improvements in this area.

Dermatologists recommended ‘no further action’ for fewer lesions in the SSC pathway cohort (56%) and 2020 VLC cohort (57%), compared to the 2016 VLC cohort (69%). They more frequently recommended surgery (by 10%), monitoring (4%), and topical treatment (3%) in the SSC pathway compared to 2016 VLC. This may reflect reduced confidence due to the inferior quality of the teledermatology images or the dermatologists’ individual practices, with only one out of three diagnosing the 2016 cohort also diagnosing in 2020. In the 2020 VLC group, the main increases were 5% increased monitoring (due to more immunosuppressed and transplant patients requiring surveillance), 6% increased topical treatment, and 2% more in-person review. The rates of surgical treatment were the same as in 2016 at 16%.

The SSC pathway dermatologist–histology diagnostic concordance for benign/malignant of 70% and the 2016 VLC of 71% are consistent with a systematic review of 21 studies in which teledermatologist-histology concordance was 51–85% [31]. There was a slight improvement in overall concordance for specific diagnoses in the SSC pathway cohort at 66% (53% complete, 13% partial), compared to 2016 VLC at 57% (57% complete, 0% partial). Partial SSC pathway concordance reflects a lack of confidence in lesions of poor quality.

One potential problem encountered when calculating concordance is the ambiguity among histopathologists, dermatologists, and GPs regarding classification of some lesions, particularly atypical naevus and melanoma-in-situ (one is benign and the other malignant) [32]. SCC-in-situ was categorised as pre-malignant and SCC as malignant, so clinician classification as the former and pathologist classification as the latter would be discordant; in practice, both diagnoses would be managed similarly. These classification differences may have increased non-concordance between dermatologist and histology.

The average age of patients in the total SSC pathway cohort was 6 years older than in the 2016 VLC cohort; this is only partially explained by an aging population in the Waikato area. Statistical data from NZ stats show the median age of the Waikato region to be 37.6 years in 2013 and 38.2 years in 2018 [33]. The convenience of the SSC referral method may encourage GPs to refer a greater proportion of older patients, as has been found in other studies [18,34,35,36]. A higher proportion of 2020 VLC referrals were from semi-rural locations compared to 2020 SSC pathway and 2016 VLC. This suggests that GPs in these locations have less access or familiarity with dermoscopy.

New Zealand Europeans made up the majority of patients referred to the SSC pathway and 2016 VLC. Only 6% and 7% of patients referred to the SSC pathway and to the 2016 VLC respectively were Maori, whereas the total proportion of Maori in the Waikato region was 21% in 2018, likely reflecting lower rates of skin cancer associated with darker skin types [33].

The dermatologists indicated that the large majority of referrals to the SSC pathway had images of adequate quality (86%); poor quality was usually associated with lack of dermoscopic images, images out of focus, and, less often, lack of macroscopic images. Other reasons for poor quality included excessive blood/hair or insufficient gel used for dermoscopy images. Melanoma diagnosis requires high quality macroscopic and dermoscopic images to diagnose confidently. A systematic review assessing the use of smartphones to detect melanoma reported that up to 20% of images were of insufficient quality for diagnosis [37]. A randomised controlled trial assessing smartphone images with dermoscopy in participants without healthcare training found 5% were inadequate for diagnosis [38].

Not all patients referred to a dermatologist with a skin cancer have had a full skin check by a competent health professional. Patients with one skin cancer are likely to have others [39,40,41]. We reviewed the small subset of patients who had a related in-person assessment by a hospital plastic surgeon or dermatologist: incidental lesions were identified in 11 patients (27%) in the matched SSC pathway cohort, 2 patients (8%) in the 2020 VLC cohort, and 22 patients (24%) in the 2016 VLC cohort with histology available for few of these lesions (11, 2, and 16 respectively). We can extrapolate that many of the patients diagnosed with skin cancer through the SSC pathway will have had other significant lesions.

Limitations: The majority of skin lesions in 2020 were assessed by the SSC pathway. Only those in which the GP did not provide images or images were deemed inadequate were seen in VLC. This cohort has a potential selection bias for lesions that were more difficult to diagnose or were more likely to be malignant that may explain a lower diagnostic concordance in 2020 VLC compared to the SSC pathway. This could potentially be an issue, to a lesser extent in the 2016 VLC, if referrers had a higher threshold for referral. Low numbers and non-significant *p*-value may also reflect the small sample size. Histology reviews were limited to biopsies requested by the dermatologists; we did not look for histology of any lesion that the teledermatologist had not indicated should be excised and thus we did not calculate a false negative rate. Some SSC referrals were missed due to misleading referral description on manual searching. Follow-up time was limited to 4 months after the 2020 cohorts, excluding 19% of the 2020 VLC cohort as they had not yet received a planned outpatient appointment or surgical procedure. This compares to 17% of the 2016 VLC cohort that did not attend. For the matched SSC pathway cohort, histology was unavailable for 27 lesions (19% of those planned for excision), compared to the 2016 VLC cohort where histology was not found for 8 lesions (6%).

## 5. Conclusions

We found that referrals for skin lesions imaged in primary care with variable devices and image quality results in faster dermatologist advice with similar diagnostic performance, compared to referrals to nurse-led specialist clinics where there is consistently high-quality imaging. Use of the SSC pathway encourages early referral and diagnosis of atypical melanocytic lesions. Detection of incidental lesions in some of the skin cancer patients seen in-person is a reminder to encourage full skin examination and a low threshold for referral of suspicious lesions. Improved diagnostic performance through the SSC pathway could be achieved by more consistent adherence to the imaging requirements of the SSC referral pathway. Our results support increased uptake of teledermatological solutions in skin lesion assessment. This will be important as support tools offering augmented intelligence are integrated into clinical practice in the future. The lack of in-person lesion evaluation by dermatologists creates a burden on primary care to undertake skin checks and identify suspicious lesions requiring a second opinion.

## Figures and Tables

**Table 1:** Patient characteristics.

Variable	Total SSC *n* = 1307 (%)	Matched SSC *n* = 481 (%)	2020 VLC *n* = 108 (%)	*p*-Value	2016 VLC *n* = 400 (%)	*p*-Value
Age:						
Overall mean (SD)	61 yr (19.2)	55 yr (21.0)	59 yr (16.1)	<0.001	55 yr (21.0)	<0.001
0–9 years	25 (2)	11 (2)	1 (1)		11 (3)	
10–19 years	35 (3)	23 (5)	1 (1)		19 (5)	
20–29 years	55 (4)	32 (7)	3 (3)		27 (7)	
30–39 years	73 (6)	45 (9)	7 (6)		33 (8)	
40–49 years	116 (9)	60 (12)	13 (12)		50 (13)	
50–59 years	216 (17)	73 (15)	26 (24)		57 (14)	
60–69 years	306 (23)	100 (21)	30 (28)		89 (22)	
70–79 years	292 (22)	86 (18)	18 (17)		78 (20)	
80–89 years	163 (12)	41 (9)	7 (6)		31 (8)	
90+ years	38 (3)	10 (2)	2 (2)		5 (1)	
Sex:						
Female	738 (56)	309 (64)	64 (59)		254 (64)	
Male	569 (44)	172 (36)	44 (41)		146 (37)	
				0.57		0.01
Ethnicity:						
New Zealand European	1096 (84)	378 (78)	80 (74)		317 (79)	
Maori	73 (6)	30 (6)	12 (11)		26 (7)	
Pasifika	12 (1)	4 (1)	1 (1)		2 (1)	
European, other	82 (6)	50 (10)	11 (10)		42 (11)	
Asian	22 (2)	14 (3)	2 (2)		9 (2)	
Other	22 (2)	5 (1)	2 (2)		4 (1)	
				0.13		0.04
Patient Location:						
Urban	770 (59)	290 (60)	37 (35)		211 (53)	
Semi-rural	481 (37)	171 (36)	64 (60)		169 (42)	
Rural	55 (4)	19 (4)	6 (6)		20 (5)	
				<0.01		0.10
Referrer location:						
Urban	798 (61)	303 (63)	35 (33)		216 (54)	
Semi-rural	473 (36)	171 (32)	66 (62)		175 (44)	
Rural	20 (2)	8 (2)	5 (5)		9 (2)	
				<0.01		0.02

SSC—suspected skin cancer pathway; VLC—virtual lesion clinic; yr—years; SD—standard deviation.

**Table 2:** Image quality for SSC lesions (*n* = 186 * (%)).

Reason for Poor Quality	Not Able to Diagnose	Able to Diagnose
No dermoscopic image	26 (37)	35 (30)
No macroscopic image	4 (6)	44 (38)
Image out of focus	15 (21)	23 (20)
Other poor quality	22 (31)	15 (13)
Dermoscopy imaging incomplete	2 (3)	3 (3)
No images	10 (14)	0
Unable to open images	2 (3)	0
Different patient’s images	1 (1)	0

SSC—suspected skin cancer pathway. * Some lesions had more than one reason for inadequate quality.

**Table 3:** Time to advice and treatment.

Variable	Total SSC *n* = 1307	Matched SSC *n* = 481	2020 VLC *n* = 108	*p*-Value	2016 VLC *n* = 400	*p*-Value
Median time from referral to dermatologist advice (SD, range)	4.0 days (2.8, 0–19)	5.0 days (2.6, 0–16)	42.0 days (29.3, 16–184)	<0.001	50.0 days (43.0, 17–313)	<0.001
Median time from referral to triage (SD, range)	N/A	N/A	2.0 days (2.2, 1–15)	<0.001	3.0 days (2.3, 2–25)	<0.001
Median wait time from referral to VLC clinic (SD, range)	N/A	N/A	26.0 days (29.9, 0–173)		43.0 days (40.0, 1–308)	
Median time from advice to definitive treatment (SD, range)	N/A	21.5 days (52.4, 0–236) *n* = 102	45.0 days (38.7, 4–142) *n* = 28	0.76	60.0 days (60.8, 2–365) *n* = 104	<0.001
Median time from referral triage to definitive treatment (SD, range)	N/A	21.5 days (52.4, 0–236)	94.0 days (48.1, 25–194)	<0.001	112.0 days (68.0, 30–378)	<0.001

SSC—suspected skin cancer pathway; VLC—virtual lesion clinic; SD—standard deviation; N/A—not applicable.

**Table 4:** Lesion classification.

Variable	Matched SSC				
	Dermatologist Diagnosis *n* = 528 (%)	GP Diagnosis *n* = 548 (%)	*p*-Value	Histological Diagnosis *n* = 113 (%)	*p*-Value
Benign	343 (65)	237 (43)		32 (28)	
Pre-malignant	52 (10)	19 (4)		5 (4)	
Malignant	116 (22)	181 (33)		76 (67)	
Uncertain	17 (3)	111 (20)		N/A	
			<0.001		<0.001
Benign:malignant	3.0	1.3		0.4	
	2020 VLC				
	Dermatologist Diagnosis *n* = 277 * (%)	GP diagnosis *n* = 172 (%)	*p*-Value	Histology Diagnosis *n* = 33 (%)	*p*-Value
Benign	177 (64)	30 (17)		11 (33)	
Pre-malignant	40 (14)	11 (6)		2 (6)	
Malignant	52 (19)	18 (11)		20 (61)	
Uncertain	8 (3)	113 (38)		N/A	
			<0.001		0.09
Benign:malignant	3.4	1.7		0.6	
	2016 VLC				
	Dermatologist Diagnosis *n* = 680 * (%)	GP Diagnosis *n* = 603 (%)	*p*-Value	Histology Diagnosis *n* = 122 (%)	*p*-Value
Benign	460 (67)	149 (25)		21 (17)	
Pre-malignant	65 (10)	16 (3)		14 (11)	
Malignant	121 (18)	183 (30)		87 (71)	
Uncertain	34 (5)	255 (42)		N/A	
			<0.001		<0.001
Benign:malignant	3.8	0.8		0.2	

SSC—suspected skin cancer pathway; VLC—virtual lesion clinic; GP—general practitioner; N/A—not applicable; IEC—intraepithelial carcinoma; SCC—squamous cell carcinoma; BCC—basal cell carcinoma. * Includes extra lesions identified by nurse specialist during VLC.

**Table 5:** Histology lesion classification.

Variable	Matched SSC *n* = 113	2020 VLC *n* = 33	2016 VLC *n* = 122
Keratinocytic:melanocytic	1.8	1.2	3.4
Total number MIS	22	6	14
Total number melanoma	6	3	6
MIS:melanoma	3.7	2.0	2.3

SSC—suspected skin cancer pathway; VLC—virtual lesion clinic; MIS—melanoma-in-situ.

**Table 6:** Lesion advice.

Variable	Matched SSC *n* = 528 (%)	2020 VLC *n* = 277 (%)	*p*-Value	2016 VLC *n* = 682 (%)	*p*-Value
No further management	298 (56)	157 (57)		471 (69)	
Monitor	38 (7)	21 (8)		18 (3)	
Topical	55 (10)	37 (13)		50 (7)	
Surgical	136 (26)	43 (16)		112 (16)	
In-person review	1 (0)	19 (7)		31 (5)	
			<0.001		<0.001

SSC—suspected skin cancer pathway; VLC—virtual lesion clinic.

**Table 7:** Diagnostic concordance.

**GP-Dermatologist Concordance**					
**Variable**	**Matched SSC** ***n* = 528 (%)**	**2020 VLC** ***n* = 172 (%)**	***p*-Value**	**2016 VLC** ***n* = 601 (%)**	***p*-Value**
Benign/malignant:					
Concordant	305 (58)	38 (22)		194 (32)	
Not concordant	223 (42)	134 (78)		407 (68)	
			<0.001		<0.001
Specific diagnosis:					
Completely concordant	183 (35)	37 (22)		101 (17)	
Partially concordant	60 (11)	16 (9)		39 (7)	
Not concordant	285 (54)	119 (69)		461 (77)	
			<0.001		<0.001
**Dermatologist-Histology Concordance**					
**Variable**	**Matched SSC** ***n* = 114 (%)**	**2020 VLC** *n*= 32 * (%)	***p*-Value**	**2016 VLC** ***n* = 112 (%)**	***p*-Value**
Benign/malignant:					
Concordant	80 (70)	20 (63)		86 (71)	
Not concordant	34 (30)	12 (38)		36 (30)	
			0.41		0.96
Specific diagnosis:					
Completely concordant	60 (53)	19 (59)		70 (57)	
Partially concordant	15 (13)	2 (6)		0 (0)	
Not concordant	39 (34)	11 (34)		52 (43)	
			0.54		<0.001
**GP-Histology Concordance**					
**Variable**	**Matched SSC** ***n* = 114 (%)**	**2020 VLC** ***n* = 17 (%)**	***p*-Value**	**2016 VLC** ***n* = 98 (%)**	***p*-Value**
Benign/malignant:					
Concordant	68 (60)	2 (12)		48 (49)	
Not concordant	46 (40)	15 (88)		50 (51)	
			<0.001		<0.001
Specific diagnosis:					
Completely concordant	46 (40)	1 (6)		40 (41)	
Partially concordant	9 (8)	1 (6)		5 (5)	
Not concordant	59 (52)	15 (88)		53 (54)	
			0.02		0.71

SSC—suspected skin cancer pathway; VLC—virtual lesion clinic. * Includes extra lesions identified by nurse specialist during VLC.

## Data Availability

Original data for this study is retained on a protected District Hospital Server. The authors have not prepared a dataset suitable for a public archive.

## Supplementary Materials

The following are available online at https://www.mdpi.com/article/10.3390/cancers13225828/s1, Table S1: Patient demographics of incidental lesions during specialist follow-up, Table S2: Incidental lesions during specialist follow-up, Table S3: Incidental lesions during VLC.

## Appendix A

**Table A1:** Diagnosis and Outcome Classification.

**Specific Diagnoses**	**Includes:**
Melanocytic naevus	Junctional naevus, dermal naevus, compound naevus, blue naevus, acral naevus, Myerson naevus, cockade naevus, other benign naevus
Atypical naevus	Atypical naevus, Spitz naevus, Reed naevus
Benign keratosis	Seborrhoeic keratosis, solar lentigo, lichen planus-like keratosis, porokeratosis, viral wart
Angioma	Angioma
Dermatofibroma	Dermatofibroma
Actinic keratosis	Actinic keratosis, actinic cheilitis
IEC	Intraepithelial carcinoma
SCC	Squamous cell carcinoma, keratoacanthoma, cutaneous horn
BCC	Nodular basal cell carcinoma, morphoeic basal cell carcinoma, pigmented basal cell carcinoma, recurrent basal cell carcinoma, superficial basal cell carcinoma
Melanoma	Superficial spreading melanoma, nodular melanoma, acral melanoma, lentigo maligna melanoma, melanoma-in-situ, lentigo maligna.
Other	All other lesions not covered in the above diagnoses
**Benign–Malignant Classification**	**Includes:**
Benign	Melanocytic naevus, atypical naevus, benign keratosis, angioma, dermatofibroma, benign lesions in the other category
Malignant	Melanoma, BCC, SCC
Pre-malignant	IEC, actinic keratosis
Uncertain	Lesions labelled uncertain or diagnosis not provided
**Outcome Advice**	**Includes:**
No action	No further lesion assessment or treatment required
Monitor	GP or VLC skin lesion monitoring
Topical	Cryotherapy, 5-fluorouracil, or imiquimod treatment
Surgical	Excision or biopsy
Review	Face-to-face review

IEC—intraepidermal carcinoma; SCC—squamous cell carcinoma; BCC—basal cell carcinoma; VLC—virtual lesion clinic.
