# Supplementary material for: Remote Skin Cancer Diagnosis: Adding Images to Electronic Referrals Is More Efficient Than Wait-Listing for a Nurse-Led Imaging Clinic
Source: Cancers (Basel). 2021 Nov 20;13(22):5828. doi: 10.3390/cancers13225828 (PMC8616500; doi:10.3390/cancers13225828)
Supplement: Supplementary file 1 [file cancers-13-05828-s001.zip › cancers-1422429-supplementary.pdf]

cancers

MDPI

Supplementary Materials: Remote Skin Cancer Diagnosis: Adding Images to Electronic Referrals Is More Efficient than Wait-Listing for a Nurse-Led Imaging Clinic

Leah Jones, Michael Jameson, and Amanda Oakley

Table S1. Patient demographics of incidental lesions during specialist follow-up

Variable	Matched SSC *n* = 41	2020 VLC *n* = 24	*p*-Value	2016 VLC *n* = 91	*p*-Value
Mean age (SD)	63 yrs (15.1)	62 yrs (15.9)	0.96	67 yrs (14.4)	0.26
		Gender: N (%)			
Female	26 (59)	14 (58)		52 (57)	
Male	18 (41)	10 (42)		39 (43)	
			<0.001		0.83
		Ethnicity: N (%)			
New Zealand European	40 (91)	20 (83)		80 (88)	
Maori	0 (0)	0 (0)		2 (2)	
Pacific	1 (2)	0 (0)		0 (0)	
Other European	2 (5)	2 (8)		8 (9)	
Asian	1 (2)	0 (0)		1 (1)	
Other	0 (0)	2 (8)		0 (0)	
			0.26		0.40

SSC—suspected skin cancer pathway; VLC—virtual lesion clinic; SD—standard deviation.

Table S2. Incidental lesions during specialist follow-up

Variable	Matched SSC *n* = 41	2020 VLC *n* = 24	*p*-Value	2016 VLC *n* = 91	*p*-Value
Total number (%) of incidental lesions identified	11 (27)	2 (8)		22 (24)	
Mean (SD) incidental lesions per patient	0.2 (0.6)	0.0 (0.3)	0.64	0.2 (0.7)	0.95
	**Specialist specific diagnosis**				
Actinic keratosis	1 (1)	0 (0)		3 (14)	
SCC	1 (1)	0 (0)		3 (14)	
BCC	4 (36)	1 (50)		14 (64)	
Uncertain	5 (45)	1 (50)		2 (9)	
			0.93		0.09
Lesions with histology available	4	2		16	
	**Benign/malignant lesion classification**				
Benign	1 (1)	1 (50)		7 (42)	
Pre-malignant	2 (18)	0 (0)		3 (19)	
Malignant	8 (73)	1 (50)		6 (38)	
			0.18		0.12
BMR	0.1	1		1.2	
Keratinocytic:melanocytic	3.0	1.0		1.0	
MIS:melanoma	2	0		0	

SSC—suspected skin cancer pathway; VLC—virtual lesion clinic; SD—standard deviation; yr—years; BMR—benign to malignant ratio; MIS –melanoma-in-situ.

Table S3. Incidental lesions during VLC

Variable	2020 VLC *n* = 108 (%)	2016 VLC *n* = 400 (%)
Total number of additional lesions identified	29 (27)	78 (20)
Lesions with histology available	4	26
Benign/malignant lesion classification		
Benign	0 (0)	4 (15)

Pre-malignant	0 (0)	3 (12)
Malignant	4 (100)	19 (73)
Benign:malignant	0.0	0.2
Keratinocytic:melanocytic	0.3	5.3
MIS:melanoma	0.5	2.0

VLC—virtual lesion clinic; yr—years; MIS—melanoma-in-situ

Copyright: © 2021 by the authors. Licensee MDPI, Basel, Switzerland. This article is an open access article distributed under the terms and conditions of the Creative Commons Attribution (CC BY) license (https://creativecommons.org/licenses/by/4.0/).
